# Molecular markers in cell cycle visualisation during development and stress conditions in *Arabidopsis thaliana*

**DOI:** 10.1017/qpb.2024.18

**Published:** 2024-12-12

**Authors:** Olivia S. Hazelwood, M. Arif Ashraf

**Affiliations:** 1 Department of Biology, Howard University, Washington, DC, USA

**Keywords:** abiotic stress, cell biology, cell cycle, plant development, quantitative cell biology

## Abstract

Plant growth and development are tightly regulated by cell division, elongation, and differentiation. A visible plant phenotype at the tissue or organ level is coordinated at the cellular level. Among these cellular regulations (cell division, elongation and differentiation), cell division in plants follows the same universal mechanisms across kingdoms of life, and involves conserved cell cycle regulatory proteins (cyclins, cyclin-dependent kinase and cell cycle inhibitors). Cell division is regulated through distinct cell cycle steps (G1, S, G2 and M), and these individual steps are visualised using transgenic marker lines. As a result, a quantitative cell cycle approach in plants during development and stress conditions relies on the accuracy of cell cycle markers. In this perspective article, we highlight the available cell cycle marker lines in plants, common practices within plant biology communities based on existing literature and provide a road map to a thorough quantitative approach of cell cycle regulation in plants.

## Graphical abstract

**Figure:**
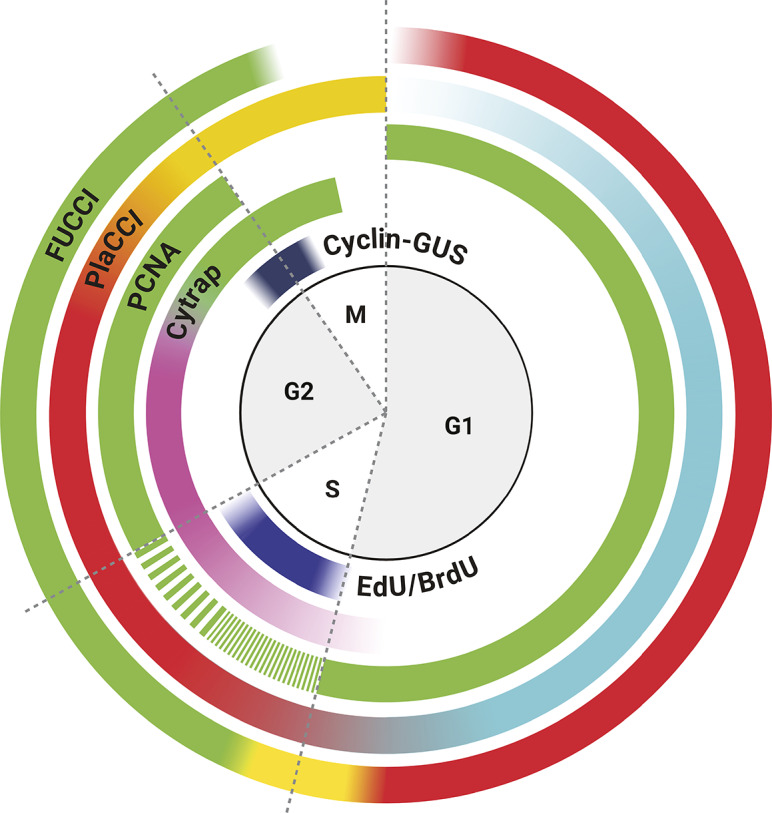

## 1. Introduction

The discovery of the microscope and first observation of the cell by Robert Hooke demonstrated that life is fundamentally cellular (Wollman, Nudd, Hedlund, & Leake, 2015). This revealed that tissues are composed of clusters of cells, raising the question: From where are all these cells coming from? In the 1800s, cell theory suggested that all cells come from other pre-existing cells (Wolpert, 1996). The theory was experimentally proven by Walther Flemming, who used textile dye to observe that a mother cell divides to produce two new daughter cells (Wolpert, 1996). In the later part of 1900s, we made major progress in understanding cell division through genetic, biochemical and cell biology studies pioneered by Leland Hartwell, Timothy Hunt, and Paul Nurse, who was awarded the Nobel Prize in Physiology and Medicine in 2001 (Nurse, 2000; Reid, Culotti, Nash, & Pringle, 2015; Uzbekov & Prigent, 2022). Their studies highlighted that cell division follows a tightly regulated cycle, where (a) DNA is synthesised and doubles in number, known as the synthesis or S phase, (b) the genetic material is divided into two new cells during mitosis or M phase; (c) these major steps are separated in time by two gap phases (G1 and G2) (Nurse, 2000; Reid et al., 2015; Uzbekov & Prigent, 2022). Each of these steps is regulated by cyclins, cyclin-dependent kinases and cell cycle inhibitors (Shimotohno, Aki, Takahashi, & Umeda, 2021). These major cell cycle regulators are conserved across the kingdom (Shimotohno et al., 2021). The importance of cell cycle regulation is observed during organismal development, and the disturbance of cell cycle regulation is observed during human diseases and organismal response to environmental cues.

Due to the fundamental role of the cell cycle in development, disease and environmental response in organisms, it has become increasingly important to visualise and quantify the cell cycle status in both a spatial and temporal manner. With the advent of fluorescent protein tagging and microscopic imaging, we can take advantage of cell cycle regulators which appear and disappear in specific cell cycle phases. For instance, cell cycle marker like FUCCI (*F*luorescent, *U*biquitination-based *C*ell *C*ycle *I*ndicator) allows visualisation of G1, G1/S and S/G2/M in animal cells by utilising cell cycle regulators Cdt1 (cyclin-dependent kinase type1) and Geminin (Sakaue-Sawano et al., 2008). Both Cdt1 and Geminin are under the control of ubiquitin-mediated proteolysis by SCF^Skp2^ and APC^Cdh1^ complexes, respectively (Benmaamar & Pagano, 2005; Sakaue-Sawano et al., 2008; Wei et al., 2004). These two proteolytic complexes act upon the corresponding substrates in a cell cycle-dependent manner. For instance, SCF^Skp2^ and APC^Cdh1^ complexes are active in late M to G1 and S to G2 phases, respectively. Additionally, SCF^Skp2^ and APC^Cdh1^ complexes inhibit each other in a reciprocal manner during the cell cycle (Benmaamar & Pagano, 2005; Sakaue-Sawano et al., 2008; Wei et al., 2004). As a result, Cdt1 fluoresces red in the G1 phase, Geminin fluoresces green in the S/G2/M phases, and the intermediate G1/S fluoresces yellow. Altogether, FUCCI allows fluorescent cell sorting and live-cell imaging to track and quantify distinct cell cycle stages.

**Figure 1.**
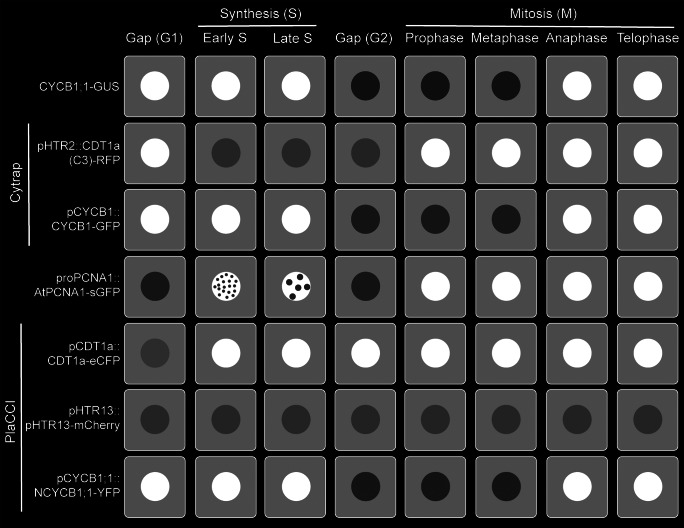
Visualisation of plant cell cycle marker lines (CYCB1;1-GUS, Cytrap, proAtPCNA1::AtPCNA1-sGFP and PlaCCI) throughout the distinct cell cycle stages (G1, early S, late S, G2, prophase, metaphase, anaphase and telophase).

Ideally, it would be convenient to fluorescently tag Cdt1 and Geminin to generate cell cycle marker lines in other species. Unfortunately, both Cdt1a and Geminin are not universally conserved. For instance, plants and yeast lack the Geminin gene, limiting the use of a FUCCI-like system (Caro, Castellano, & Gutierrez, 2007; Caro & Gutierrez, 2007; Desvoyes, Arana-Echarri, Barea, & Gutierrez, 2020). As a result, plant cell biologists have tried different approaches to develop comprehensive cell cycle markers, and the contributions to this endeavour from different labs over the last few decades has created a compelling story (Colón-Carmona, You, Haimovitch-Gal, & Doerner, 1999; Desvoyes et al., 2020; Yin et al., 2014; Yokoyama, Hirakawa, Hayashi, Sakamoto, & Matsunaga, 2016). A recent review on plant cell cycle marker lines took a holistic approach to highlight chemical labelling, constitutively expressed nuclear markers, cell cycle phase-specific markers and combined reporters for plants (Echevarría, Gutierrez, & Desvoyes, 2021). In this perspective article, we are focusing on four major cell cycle marker lines (CYCB1;1-GUS, Cytrap, proAtPCNA1::AtPCNA1-sGFP and PlaCCI), because these are (1) examples of how plant biologists are constantly innovating to create a comprehensive cell cycle marker and (2) widely used by the plant biology community for developmental and environmental stress studies. Additionally, we will highlight examples of current quantitative cell cycle approaches, challenges and provide a guideline for a comprehensive quantitative approach.

*Plant cell cycle markers:* In the following section, we are going to highlight four major cell cycle markers (CYCB1;1-GUS, Cytrap, proAtPCNA1::AtPCNA1-sGFP and PlaCCI) available in the model plant *Arabidopsis thaliana*. For each of these marker lines, we will chronologically discuss the development, advantages and challenges for quantitative cell cycle approaches in each one of these marker lines.

*CYCB1;1-GUS:* This marker line utilised a fundamental property of mitotic cyclins, which is that they peak around the end of G2 and degrade at the end of M phase (Figure 1). Due to its precise temporal regulation during cell cycle, mitotic cyclins are appropriate genes for cell cycle markers. Using this principle, Colón-Carmona et al. developed a Cyclin-GUS marker that could reliably visualise mitotic activity occurring during the G2/M phase transition (Colón-Carmona et al., 1999). This marker line contains the *AtCYCB1;1* promoter region (1148 bp), mitotic destruction box (N-terminal 116 amino acids region including the peptide [RQVLGDIGN] responsible for destruction of CYCB1;1) and fused to the β-glucuronidase (GUS) reporter. The efficiency of CYCB1;1-GUS as a G2/M marker was tested through RNA blot analysis and histochemical assay using tobacco BY-2 (Bright Yellow-2) cell line and the model plant *Arabidopsis thaliana* (Colón-Carmona et al., 1999). To date, the CYCB1;1-GUS marker line original article has been cited 726 times, clearly demonstrating the importance of this marker line within the community. Later on, GUS was replaced by mGFP5 to create the CYCB1;1-GFP by Malcolm Bennett’s group (Ubeda-Tomás et al., 2009).

Researchers have used CYCB1;1-GUS marker line to reliably identify actively dividing cells in developmentally important mutant background and in stress conditions. Due to the large number of references available, it is impossible to comprehensively assess the applications of the CYCB1;1-GUS marker line. Instead, we will highlight a few examples involving the CYCB1;1-GUS marker line that demonstrate its use in both developmental and stress conditions. In development, a histochemical GUS assay in the NUCLEOSTEMIN-LIKE1 (NSN1) mutant (*nsn1*) indicated that the number of actively dividing cells were less in number in both the shoot apical meristem and root apical meristem (Wang, Wang, Xie, Hong, & Yang, 2018). In stress conditions, Arabidopsis root growth is inhibited at 4°C. Using the CYCB1;1-GUS marker line, Ashraf et al. demonstrated that cold stress inhibits only cell division, and not cell elongation (Ashraf & Rahman, 2019). This study also quantified the GUS activity from histochemical images and provided a guideline for quantification-based CYCB1;1-GUS imaging (Ashraf & Rahman, 2019). The use of CYCB1;1-GUS during stress conditions should be considered with caution, because the expression of CYCB1;1 increases during certain stress conditions, such as DNA damage (Schnittger & De Veylder, 2018). As a result, the CYCB1;1-GUS data during stress conditions should be re-confirmed with another independent cell cycle marker.

*Cell cycle tracking in plant cells (Cytrap):* CYCB1;1-GUS marker line indicates the actively dividing cells in a tissue. But it does not provide information about the prior cell cycle stages (G1, S and G2). An alteration in the number of actively dividing cells may occur due to G2/M phase specific regulators or as a consequence of earlier cell cycle regulators. The quantitative information of the rest of the cell cycle remains unknown when only the CYCB1;1-GUS marker line is used.

To solve this problem, Masaaki Umeda’s lab developed a dual-colour marker, known as *C*ell *Cy*cle *Tra*cking in *P*lant cells (Cytrap; mentioned as Cytrap in the remaining article) (Yin et al., 2014; Figure 1). This marker line utilises *pHTR2::CDT1a (C3)-RFP* and *pCYCB1::CYCB1-GFP*, previously reported and published by Ubeda-Tomás et al. (2009), to visualise the S/G2 phase and G2/M phase, respectively. The Arabidopsis homologue of yeast CDT1 (cyclin-dependent kinase type1), CDT1a, is an essential protein to indicate the origins of DNA replication, and is exported or proteolysed in other eukaryotes after DNA replication (Nishitani, Lygerou, Nishimoto, & Nurse, 2000; Nishitani, Taraviras, Lygerou, & Nishimoto, 2001). This characteristic of the CDT1a protein makes it an ideal candidate for an S-phase marker. As prior proof of concept, Cdt1 was used as a G1-/S-phase indicator in the FUCCI marker (Sakaue-Sawano et al., 2008). To avoid undesirable phenotypic alterations, a non-functional C-terminal fragment of *CDT1a* (mentioned as C3, includes 363–571 amino acids or 1578–2505 genomic regions) was used to fuse with GFP instead of the entire coding region. Because the fluorescent intensity of *CDT1a (C3)-GFP* was weak during initial imaging, Yin et al. replaced the CDT1a promoter with an S-phase-specific promoter, *HISTONE THREE RELATED2 (HTR2*) (1.1 kbp of promoter region) based on the previously published expression data of histone H3 family in Arabidopsis (Okada, Endo, Singh, & Bhalla, 2005). *pHTR2::CDT1a (C3)-RFP* was introduced into *pCYCB1::CYCB1-GFP* transgenic line background (Figure 1). Additionally, cells without a RFP or GFP signal indicate the G1 phase.

Cytrap provides a good quantitative approach for cell cycle progression. For instance, the root growth of the model plant Arabidopsis is reduced in dark conditions. Using Cytrap marker, Geoffrey Wasteneys’s lab demonstrated that both RFP (S/G2) and GFP (G2/M) signal disappear in dark-grown roots, indicating a stagnant cell cycle (Halat, Gyte, & Wasteneys, 2020). As an example of Cytrap’s application under stress conditions, Masaaki Umeda’s group used Cytrap during heat stress and found a decrease in the number of cells at S/G2 phase, along with an increase in cells at G2/M phase (Takahashi et al., 2019). Both examples highlight the power of dual-colour cell cycle marker lines.

*proPCNA1::PCNA1-sGFP:* Although Cytrap is an excellent comprehensive marker to quantify cells at different cell cycle stages (S/G2 and G2/M), it is difficult to measure the duration of the S phase. During that time, the S phase was visualised by staining using a thymidine analog (5-ethynyl-2’-deoxyuridine, also known as EdU), which requires fixed cells. To overcome this issue and enable quantitative imaging of DNA synthesis and S-phase duration using live-cell imaging, Yokoyama et al. developed a plant cell cycle marker targeting a protein called proliferating cell nuclear antigen (PCNA) (Yokoyama et al., 2016; Figure 1).

PCNA is an essential cofactor of DNA polymerases and act as a sliding clamp for DNA polymerase during DNA synthesis in the S phase (Maga & Hübscher, 2003; Moldovan, Pfander, & Jentsch, 2007; Yokoyama et al., 2016). In living fission yeast *Schizosaccharomyces pombe*, PCNA expression indicates the replication foci in a spatial and temporal manner during the S phase (Meister, Taddei, Ponti, Baldacci, & Gasser, 2007). Expression of AtPCNA1 fused with sGFP under the native promoter (proAtPCNA1) demonstrates three distinct expression patterns – whole, dotted and speckled – throughout the cell cycle (Yokoyama et al., 2016; Figure 1). To determine the accuracy of AtPCNA1 at reporting cell cycle phases, *AtPCNA1* seedlings were introduced to the thymidine analog EdU. This demonstrated that the three distinct fluorescent patterns – whole, dotted, and speckled – corresponded to the G1/G2, early S phase, and late S phase, respectively (Yokoyama et al., 2016; Figure 1). *proPCNA1::AtPCNA1-sGFP* (mentioned as PCNA-GFP in the remaining article) has been demonstrated to provide reliable visualisation and quantification of the G1/G2, early S and late S phases of the cell cycle (Figure 1).

PCNA-GFP plays a phenomenal role in quantitative cell cycle approaches for plant cell biologists. For instance, Masaki Ito’s group utilised the PCNA-GFP marker in the SCL28 (*S*CARE*C*ROW-*L*IKE28) mutant (*scl28*) and overexpression (*SCL28^OE^*) background. They have observed an increase in the number of G1 and early S-phase cells in the *slc28* mutant, and an increase in number of early S- and late S-phase cells in *SCL28^OE^* background (Nomoto et al., 2022).

As for stress conditions, during low temperature stress, CYCB1;1-GUS indicates a reduced number of cells at the G2/M phase (Ashraf & Rahman, 2019). But it only indicates the reduction of cell number at G2/M phase, not in the earlier phases of cell cycle. Using PCNA-GFP marker during low temperature stress, Ashraf et al. demonstrated that more cells are trapped at the gap phases (G1/G2), and consequently less number of cells are at the actively dividing stage (Ashraf & Rahman, 2019). These applications of PCNA-GFP in both developmental and stress studies demonstrate its powerful quantitative advantages.

*Plant cell cycle indicator (PlaCCI):* CYCB1;1-GUS, Cytrap and PCNA-GFP incrementally helped to visualise and quantify the plant cell cycle. But the plant cell cycle field still lacked a comprehensive marker like FUCCI. This issue has been circumvented by combining three reporters: *pCDT1a::CDT1a-eCFP* for G1 phase visualisation, *pHTR13::pHTR13-mCherry* for S and early G2 phase visualisation and *pCYCB1;1::NCYCB1;1-YFP* for late G2 and early M phase (prophase and metaphase) visualisation (Desvoyes et al., 2020) (Figure 1). This marker line is known as *Pla*nt *C*ell *C*ycle *I*ndicator (PlaCCI; and mentioned as PlaCCI for the rest of the article) and is considered as equivalent to the FUCCI marker line. PlaCCI allows detailed visualisation and quantification of cell cycle stages in the lateral root cap, shoot apical and floral meristem, developing trichomes and the petiole of developing primordia.

Furthermore, PlaCCI is the first tri-colour (relies on eCFP, YFP and mCherry) cell cycle marker line developed in plants (Desvoyes et al., 2020). PlaCCI, compared to Cytrap, has all three markers into the single construct and this approach makes it convenient for researcher to avoid the segregation issue.

To date, PlaCCI is the most comprehensive cell cycle marker for visualisation and quantification in plants, offering a more advanced quantitative approach than ever before. Dior Kelley’s lab utilised the PlaCCI marker in the Arabidopsis thaliana MYOSIN1 (ATM1) mutant (*atm1-1*) background and found that S-phase entry is defective (Olatunji, Clark, & Kelley, 2023). Furthermore, in a stress condition, such as Zeocin-induced DNA damage, a higher number of cells accumulated at the G1 phase based on the CDT1a-eCFP of the PlaCCI marker line (Kutashev et al., 2024). This study also demonstrated that increased number of G1 cells is correlated with incremental Zeocin concentration (Kutashev et al., 2024). Both examples – one in a developmental mutant context and the other under stress conditions – demonstrated the quantitative advantages of using the PlaCCI marker line.

**Figure 2.**
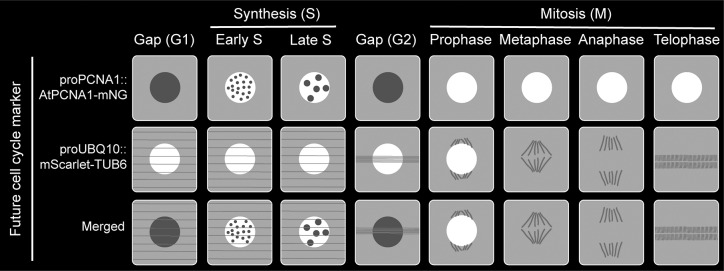
Proposed plant cell cycle marker line combining proAtPCNA1::AtPCNA1-mNeonGreen and proUBQ10::mScarlet-TUB6 to visualise distinct cell cycle stages (G1, early S, late S, G2, prophase, metaphase, anaphase and telophase) and mitotic structures.

*Future of quantitative cell cycle approaches in plants:* Despite the lack of the Geminin gene in plants and challenges in development of a FUCCI-like marker, plant cell biologists took series of innovative approaches to develop cell cycle marker lines. But there are still more challenges and room for improvement in the near future.

If we look carefully at each of the widely used markers by the plant biology community, it indicates that the G1 phase does not have any detectable markings in the Cytrap marker. Several published articles took advantage of this missing fluorescent G1 phase as a quantitative approach (Takahashi et al., 2019). Detecting the early G1 phase is also challenging using the PlaCCI marker, because CDT1a-eGFP expression requires time to reach to a detectable level (Desvoyes et al., 2020). The G1 phase is visible through a ‘whole’ nuclear expression pattern of PCNA-GFP (Yokoyama et al., 2016). But both gap phases, G1 and G2, have a similar ‘whole’ nuclear pattern (Figure 1). An alternative way of distinguishing between G1 and G2 phase is comparing the nuclear area, where late G2 possess larger nuclear area than G1 phase (Chen et al., 2017).

In the last several decades, after making tremendous progress in developing a comprehensive cell cycle marker, none of the existing markers are perfect, and each one of them has its own limitations and challenges. Furthermore, the individual mitotic steps (prophase, metaphase, anaphase, telophase and cytokinesis) are not distinguishable using the existing cell cycle marker lines (Figure 1). However, a fluorescently tagged tubulin line is effective for observing the cortical microtubule array, preprophase band, spindle and phragmoplast, thereby visualising the different mitotic structures and stages (Allsman et al., 2023; Buschmann, Holtmannspötter, Borchers, O’Donoghue, & Zachgo, 2016). Based on our current understanding and working experience, combining PCNA-GFP with a tubulin marker such proUBQ10::mRFP-TUB6 will be a better combination to visualise cell cycle stages along with individual mitotic stages (Figure 2). Furthermore, brighter and monomeric fluorophores such as mNeonGreen and mScarlet can be used instead of GFP and mRFP, respectively. Additionally, both PCNA and tubulin genes are conserved across the plant kingdom. Therefore, the successful strategy of combining PCNA and tubulin can be applied to transform various plant species.

The final major challenge involves the quantification of cell cycle marker images. Currently, the plant biology community relies on manually counting and sorting each cell to the cell cycle stages. A good image segmentation, more precisely nuclear segmentation, will help to make quantification less labour intensive and time efficient. Currently, a whole array of nuclear segmentation tools are available and already in use for different organisms, including plant systems in many aspects (Caicedo et al., 2019; Lee et al., 2022; Lin, Chung, & Tan, 2023). Creating an image analysis platform, training the algorithm with existing plant cell cycle marker images, and launching it with an intuitive, user-friendly interface will advance plant cell cycle quantification to the next level.
