# Supplementary material for: Molecular markers in cell cycle visualisation during development and stress conditions in Arabidopsis thaliana
Source: Quant Plant Biol. 2024 Dec 12;5:e14. doi: 10.1017/qpb.2024.18 (PMC11706682; doi:10.1017/qpb.2024.18)
Supplement: Peer review history

# Editor report: Recommendation: Molecular markers in cell cycle visualisation during development and stress conditions in *Arabidopsis thaliana*— R0/PR2

**DOI:** 10.1017/qpb.2024.18.pr2

**Affiliations:** Graduate School of Life Sciences, Tohoku University, Japan

The referee comments were positive overall and we would like to consider your paper further. However, you need to first improve at all the points commented by the reviewers and revise your manuscript accordingly before your paper can be considered acceptable for publication.

# Editor report: Decision: Molecular markers in cell cycle visualisation during development and stress conditions in *Arabidopsis thaliana*— R0/PR3

**DOI:** 10.1017/qpb.2024.18.pr3

**Affiliations:** RDP, ENS de Lyon, France

No accompanying comment.

# Editor report: Recommendation: Molecular markers in cell cycle visualisation during development and stress conditions in *Arabidopsis thaliana*— R1/PR5

**DOI:** 10.1017/qpb.2024.18.pr5

**Affiliations:** Graduate School of Life Sciences, Tohoku University, Japan

No accompanying comment.

# Editor report: Decision: Molecular markers in cell cycle visualisation during development and stress conditions in *Arabidopsis thaliana*— R1/PR6

**DOI:** 10.1017/qpb.2024.18.pr6

**Affiliations:** RDP, ENS de Lyon, France

No accompanying comment.
